# Physical and Quality of Life Changes in Elderly Patients after Laparoscopic Surgery for Colorectal Cancer—A Prospective Cohort Study

**DOI:** 10.3390/ijerph192214711

**Published:** 2022-11-09

**Authors:** Rochelle Mey, José Casaña, Óscar Díaz-Cambronero, Luis Suso-Martí, Ferran Cuenca-Martínez, Guido Mazzinari, Rubén López-Bueno, Lars L. Andersen, Laura López-Bueno, Francisco Selva-Sarzo, Joaquín Calatayud

**Affiliations:** 1 Exercise Intervention for Health Research Group (EXINH-RG), Department of Physiotherapy, University of Valencia, 46010 Valencia, Spain; 2 VUMC School of Medical Sciences, Amsterdam UMC, 1105 Amsterdam, The Netherlands; 3 Department Anesthesiology, Hospital Universitari i Politécnic la Fe, 46010 Valencia, Spain; 4 Perioperative Medicine Research Group, Biomedical Research Institute la Fe, 46010 Valencia, Spain; 5 National Research Centre for the Working Environment, 2100 Copenhagen, Denmark; 6 Department of Physical Medicine and Nursing, University of Zaragoza, 50009 Zaragoza, Spain; 7 Sport Sciences, Department of Health Science and Technology, Aalborg University, 9220 Aalborg, Denmark; 8 Department of Physiotherapy, University of Valencia, 46010 Valencia, Spain

**Keywords:** physical fitness, handgrip, lower limb isometric strength, cancer, laparoscopic surgery

## Abstract

Background—The incidence of colorectal cancer is increasing among elderly people, where postoperative complications are frequent. Methods—We evaluated postoperative physical and quality of life changes in elderly patients undergoing laparoscopic surgery for colorectal cancer. A prospective cohort study was performed in 31 colorectal cancer patients ≥60 years who were scheduled for laparoscopic surgery due to colorectal cancer. Outcomes were measured one month preoperative (T1), three days postoperative (T2) and one month postoperative (T3). Results—The largest early postoperative (from T1 to T2) declines were observed for isometric knee extension strength (33.1%), 30 s Chair Stand Test (27.9%) and handgrip strength (16.9%). Significant reductions in quality of life measured with the QLQ-C30 summary score and the EQ 5D index score were found between T1–T3 and T1–T2, respectively. Conclusions—A decline in isometric knee extension strength, 30 s Chair Stand Test, handgrip strength and quality of life is evident in elderly patients in the days following laparoscopic surgery for colorectal cancer. Preoperative values are recovered one month after surgery for all the outcomes, except for isometric knee extension, which should receive especial attention.

## 1. Introduction

Colorectal cancer is the third most common cancer and fourth deadliest worldwide [1], and over the years, the risk of developing colorectal cancer will increase due to different factors, such as the sedentary lifestyle of the modern world [2,3]. Most people who are diagnosed with colorectal cancer are over the age of 60 and at high risk for frailty. Surgery is the main treatment for colorectal cancer, and postoperative complications are more frequent in the elderly population [2,4,5] due to their reduction in functional capacity and therefore physical fitness [6]. In fact, it has been shown recently that physical fitness might be a more important factor for adverse postoperative events than age, due to the complexity of the health status of the elderly [6,7].

Previous research has shown that there is an inverse association between preoperative physical fitness and severity of postoperative complications in patients with different types of cancer or treatment [8,9,10]. Focused on elderly patients with colorectal cancer undergoing surgery, a previous study showed that physically active individuals have a faster self-assessed physical recovery [11]. Accordingly, a retrospective study indicated that a higher level of physical fitness was related to a lower odds ratio for postoperative complications [12]. Recently, Karlsson et al. [13,14] performed two prospective cohort studies in elderly patients who underwent abdominal cancer resection. The authors reported that better preoperative physical fitness reduced the odds for higher complication severities, discharging to further care after surgery [13] or having limited mobility [14]. Furthermore, they showed that surgery particularly affected walking distance and functional leg strength [14]. 

The above studies have shown the relevance of preoperative physical fitness for outcome after surgery; however, none are specifically focused on elderly patients with colorectal cancer who will undergo laparoscopic surgery. This can be a relevant factor for further investigation since it has been shown that in comparison to open surgery, patients who underwent laparoscopic surgery have decreased postoperative complications, a shorter hospital stay and a faster recovery [15,16,17], something which may require a different preoperative management approach. Additionally, more research is needed to better describe which specific physical fitness components are crucial for postoperative recovery [18]. For instance, while lower limb muscle strength has been typically assessed with only one functional test [13,14] which largely reflects muscle endurance [19] and mainly involves the quadriceps muscle, a more complete and precise evaluation is needed to design optimal preoperative programmes for key muscle groups. Such knowledge is important for targeting rehabilitative programs specifically to this group of patients. 

Full recovery of the patients is not reached at hospital discharge but is achieved once they regain their daily life functions and need to be as soon as possible in order to prevent further reduction in terms of physical fitness as well as quality of life [6,20]. However, it has been shown that the quality of life of cancer patients is still impaired after treatment compared to healthy individuals [21]. The time it takes to return to participating in daily activities is dependent on the decline in physical fitness [20]. Even though it is expected to see a reduction in physical capacity after surgery, it is important to know to which extent and for which specific components of physical capacity this decline can be seen most of all [4,6]. For the purpose of reducing and preventing the risks of possible adverse outcomes after surgery, it could be important to recognize at an early stage which patients have a higher risk for developing postoperative complications. Therefore, the main objective of this study was to evaluate changes in physical and quality of life in elderly patients after laparoscopic surgery for colorectal cancer. We hypothesized that of all the physical fitness variables measured, lower limb strength would show the greater decline.

## 2. Materials and Methods

### 2.1. Subjects and Setting

A prospective observational and feasibility study was performed at the “Hospital Universitari i Politècnic La Fe” (Valencia, Spain). The study was approved by the ethical committee in Hospìtal Universitari I Politécnic la Fe, Valencia, Spain (2019-226-1) and adheres to the Strengthening the Reporting of Observational Studies in Epidemiology (STROBE) guidelines. Patients scheduled for laparoscopic surgery due to colorectal cancer were informed and asked to participate by the anesthesiologist in the preoperative visit. Medical contraindication for physical exercise was also evaluated before informed consent by the patients. Inclusion criteria for the patient population were as follows: (1): age ≥60 years; (2) scheduled for abdominal laparoscopic surgery due to colorectal cancer. The exclusion criterion was a health status that prohibits physical exercise. Prior to the baseline assessments, written informed consent was collected by the anaesthesiologist. Baseline data and preoperative physical fitness were obtained at three different time points: time point 1 (T1) is one month preoperative (+/− seven days), time point 2 (T2) is 72 h postoperative (+/− 24 h), and time point 3 (T3) is one month postoperative (+/− seven days). 

### 2.2. Demographic Data and Clinical Data

Before surgery, data on age, height, body mass index and tumor stage classification were collected from the medical records. Tumour classification was according to the Classification of Malignant Tumors (TNM), from which the T category, describing the primary tumor site and size, was included. We evaluated the health-related quality of life by using the European Organization for Research and Treatment of Cancer Quality of Life Questionnaire (QLQ-C30) [22,23], from which a summary and a global score were calculated, with higher values indicating a higher level of self-rated health and scores ranging from 30 to 126. The EQ 5D was also used to assess health-related quality of life, providing an index score, where values range from 0 to 1, and higher values denote higher health-related quality of life. 

### 2.3. Physical Fitness Measures

The Short Physical Performance Battery (SPPB), consisting of three different components: balance, four-meter gait speed and the sit-to-stand test was administered [24]. Standing balance was measured by asking the subjects to maintain a standing position for ten seconds with their feet in three different positions: side by side, semi tandem (heel of one foot next to the big toe of the other foot) and in tandem (heel of one foot directly in front of the other foot). Subjects were given a score of 1 point when they were able to hold a side-by side position for 10 s but were unable to maintain a semi-tandem position for 10 s; 2 points were given when subjects could hold a semi-tandem for 10 s but were unable to hold a tandem position for more than 2 s; 3 points were given when subjects were able to stand in full tandem position for 3 to 9 s, and 4 points were given when subjects could stand in full tandem position for 10 s. For measuring gait speed [25], subjects were asked to walk along a line of eight meters at their normal walking pace. Time was measured for walking over four meters, with two extra meters to start and two meters to stop the movement. Walking aids were allowed if necessary. The fastest time of two walks was used for scoring. Score range was from 0–4 points, with 0 points given for someone unable to walk; 1 point for a time >8.7 s; 2 points for a time between 6.21–8.7 s; 3 points for a time between 4.82–6.2 s and 4 points for a time <4.82 s. The sit-to-stand test was performed by asking the subjects to sit in a chair without armrests, cross their arms in front of their chest, and stand up from a sitting position and sit down again for five times, as fast as possible. Scoring went as follows: 1 point was given for a time ≥16.7 s; 2 points for a time between 13.7 and 16.6 s; 3 points for a time between 11.2 and 13.5 s and 4 points for a time ≤11.1 s. The sum of the three different components constituted the final score for the SPPB, ranging from 0 to 12 points, where 12 points indicated the highest degree of lower extremity functioning. 

Timed Up and Go test (TUG) [26]. This test measures the time a person takes to rise from a standard armchair, walk along a line for three meters as quickly as possible, turn around and sit down again. The person is not allowed to use their arms while standing up, but if necessary, walking aids are allowed. The highest value of two trials was used, indicating the best performance of both trials. Excellent intratester reliability has been found in elderly populations, with an intra-class coefficient of 0.94 [26]. 

Handgrip strength was assessed using a digital hand dynamometer with adjustable grip (Takei TKK5401 Grip D). Subjects were asked to stand with their arm straight down, elbow fully extended and forearm in neutral position, with their shoulder slightly abducted for approximately 10 degrees, according to previous standardized recommendations [27]. Subjects were encouraged to perform the test with maximum force for three seconds. Three trials were performed, and the highest value was used for the analysis. The TKK has shown high reliability with a very low systematic error (0.02) [27]. 

Lower limb isometric strength was analysed by performing the following different tests against fixed resistance: isometric knee flexion and extension, isometric hip abduction and adduction and ankle dorsiflexion and plantarflexion. For measuring, a portable Lafayette hand-held dynamometer was used (Nicholas Manual Muscle Tester, Lafayette Instruments). For all the trials, the dominant limb was used. Tests were performed according to the most reliable previously described techniques [28,29], as described below. For measuring the extension and flexion of the knee, subjects were seated at the examination table with their thighs in contact with the bed and their hips and knees in a constant angle of 90° [30]. In order to stabilize their position, subjects were instructed to hold on to the sides of the examination table to make sure their back stayed in an upright position. To measure extension, the dynamometer was positioned perpendicular to the tibia and fixated with straps. Flexion of the knee was measured by positioning the dynamometer on the posterior part of the lower leg. For measuring hip abduction and adduction, subjects were instructed to lie down on the examination table with their dominant leg extended and the other leg flexed [31]. For abduction, the dynamometer was fixated with straps to the table and positioned on the malleolus. Subjects were instructed to perform a maximal contraction of abduction with flexed leg. Adduction of the hip was measured in the same position with the dynamometer on the opposite side of the malleolus. For measuring isometric ankle plantar flexion and dorsiflexion, each subject was positioned in long sitting (hips flexed and knees extended) on an examination table with a backrest, according to a previous standardized procedure [32]. The assessor stabilized the lower limb proximal to the ankle joint to isolate movement at the joint and minimize additional and substitution movements. For ankle plantarflexion, this was secured against the plantar surface of the foot, just proximal to the metatarsal heads, and for ankle dorsiflexion this was secured against the dorsal surface of the foot, just proximal to the metatarsal heads. Ankle dorsiflexion and plantarflexion tests with the hand-held dynamometer have shown high reliability with ICC values ranging from 0.88 to 0.90 and 0.94 to 0.96, respectively.

All contractions were performed for three seconds, with 30 s of rest in between the trials. Three trials per movement were executed, and the mean of the three trials was used for analysis.

We also obtained the 30 s Chair Stand Test (30 s CST), where the number of chair raises within 30 s was counted [33]. For some subjects, the task was executed for only 15 s due to medical reasons, such as high blood pressure. Afterwards, level of perceived exertion was recorded according to the Borg Scale [34]. Due to the level of fatigue, this test was the last one performed. 

### 2.4. Statistical Analysis

Statistical analysis was accomplished using SPSS version 26 (SPSS Inc., Chicago, IL, USA). All variables were found to be normally distributed (Shapiro–Wilk’s normality test) before data analysis. 

Statistical comparisons for each outcome were performed using analysis of variance (ANOVA) with repeated measures. Greenhouse–Geisser correction was used when the assumption of sphericity (Mauchly’s test) was violated. Post hoc analysis with Bonferroni correction was used in the case of significant main effects. Significance was accepted at *p* ≤ 0.05.

In previous studies, it has been determined that a change in the SPPB scale of 0.4–1.5 can be considered clinically significant. This is the equivalent of the modification of one point in the SPPB. A preliminary study determined the standard deviation at 1.7 points. Therefore, for a statistical power of 90% and a significance level of 5%, the sample size in this observational study should have 31 subjects. 

## 3. Results

The included study population (*n* = 31) had a mean age of 70.9 years, and 65% were male subjects. Table 1 shows demographic characteristics at baseline. 

As presented in Table 2, declines in the different physical fitness variables were seen at the different time points. The largest declines were observed at knee extension, handgrip strength and 30 s CST. Subjects showed a significant decline in isometric strength of knee extension between T1 and T2 (Mean Difference = 7.07, *p* < 0.01) and between T1 and T3 (Mean Difference = 4.39, *p* = 0.02). A significant reduction was found in handgrip strength between T1 and T2 (Mean Difference = 4.56, *p* = 0.03). For the 30 s CST, a significant reduction was observed between T1 and T2 (Mean Difference = 4.61, *p* < 0.01), while significant increases were evident between T2 and T3 (Mean Difference = 5.13, *p* < 0.01)

Table 3 presents the differences in clinical data obtained at the different questionnaires. A significant decline was found in the QLQ-C30 summary score between T1 and T2 (Mean Difference = 10.64; *p* = 0.01). Furthermore, the EQ 5D index score was significantly impaired between T1 and T2 (Mean Difference = 0.2; *p* = 0.02) and between T2 and T3 (Mean Difference = 0.13; *p* = 0.04).

## 4. Discussion

The main results of the present study are the declines found for isometric knee extension strength (33.1%), handgrip strength (16.9%) and reduction for the 30 s CST (27.9%) between T1 and T2. Importantly, to the best of our knowledge, this is the first study providing insight into the changes in isometric lower limb strength. In addition to the decrease in physical fitness, we also found significant declines in the documented health-related quality of life. 

We hypothesized that the greatest decline would be observed in lower limb strength, due to the fact that inactivity after surgery affects the process of sarcopenia and therefore the loss of muscle mass specifically in the legs [35,36,37,38]. In line with this, we found a significant decline in the isometric lower limb strength of knee extension from T1 to T2, as well as from T1 to T3. Strength of knee extension was found to be an important factor for falling and mobility in elderly people [39]. Therefore, our findings highlight that patients might be at greater risk for falling or having lower mobility after surgery; hence, they would benefit from a preoperative training programme and an intensive and early rehabilitation programme to recover function sooner and reduce their risks of falling and improve their physical function. However, in the other components of the isometric lower limb strength, no significant declines were observed. Unfortunately, the lack of other similar studies measuring lower limb isometric strength among colorectal cancer patients or different kind of cancer patients makes comparisons difficult. 

We found a borderline significant decline in the number of repetitions of the 30 s CST from T1 to T2, while at T3 patients improved compared to preoperative and T2 values. These findings are in contrast with those found in the study conducted by Karlsson et al. [14], where the investigators reported a major short-term postoperative decline of 38%, measured 5 days postoperative. This might be explained by the older study population in the aforementioned study (mean of 76 ± 4.6 years), since age is an important factor in loss of muscle strength during bedrest [38]. Another possible explanation might be that Karlsson’s study also included patients who underwent open surgery, and a lesser decline in our study population of solely patients that underwent laparoscopic surgery would be as expected [15,16,17,40]. Furthermore, the preoperative value of our study population was considerably higher (17.4 versus 13 repetitions), which might suggest that our study population had a better functional leg strength at baseline, since a mean of 13.5 repetitions was reported as a reference value in another study in elderly subjects (mean age of 70.5 years) [33].

Our result showing a decline in handgrip strength between T1 and T2 is consistent with the finding reported in another study investigating the recovery of strength in elderly patients after major abdominal surgery, where authors found a handgrip decline of 12.1% [36]. In addition, in line with this result, another study among 140 patients reported a decline in handgrip strength three days postoperative of 9% [14]. However, in our study population, we found a stronger decline. Since handgrip strength has recently been identified as a predictive biomarker for older people for outcomes, such as general muscle strength, bone mineral density, falls and fractures, nutritional status, comorbidity, psychological factors and mortality [19], having a high handgrip strength shortly after surgery seems important for faster overall recovery. 

The SPPB is known to be a good measure for functional outcome of physical performance [35,41,42] and is related with mortality. However, we did not find any changes between the different time points in this measure. This might be due to the heterogeneity, age and size of our sample, but we cannot compare our result since, as far as we know, there are no studies using this outcome among patients with colorectal cancer undergoing laparoscopic surgery. Another important finding in our study was the consistent quality of life reduction after surgery, with declines between T1 and T3 in QLQ-C30 summary score and between T1 and T2 at the EQ 5D index score. Previous research indicated that lower QLQ-C30 is associated with more postoperative complications [43,44]. In our study population, we only found a significant reduction in QLQ-C30 after the surgery for the summary score, which is the combined score for different functional and symptom scales, and not for the global score where patients need to rate their health in general and their quality of life during the last week. Regarding the EQ 5D, it is plausible that fatigue and pain early after surgery negatively affect this outcome as shown in more advanced colorectal cancer patients [45]. In line with our result of a significant impairment of EQ 5D health-related quality of life score after surgery, another study investigating health-related quality of life in patients with CRC also found a rapid decline of the EQ 5D score after surgery [46].

The present study has some limitations. Due to the sampling method, there is still the possibility of a selection bias. Thus, we cannot be certain that the study population is representative of this patient population in general. However, our demographic data was comparatively heterogenic and therefore still a good representation of the investigated patient population, with adequate sample size. An important strength of this study was that due to the strict inclusion criteria, a very specific population was analysed. In order to provide a comprehensive description of the extent of the decline in the different components of physical fitness, we tried to give a better perspective on the relevance of specific physical fitness measures and to obtain insights needed to design optimal training programmes for decline prevention and rehabilitation. Our results indicate that functional leg strength, and specifically the movement of knee extension and handgrip strength, deserve special attention very early after surgery since these might limit daily basic physical activities. This means that the results of this study point to the need to further investigate whether targeting these outcomes in a specific training programme can improve recovery after laparoscopic surgery in elderly patients. 

## 5. Conclusions

A decline in isometric knee extension strength, handgrip strength and quality of life is evident in elderly patients after laparoscopic surgery for colorectal cancer. Preoperative values are recovered one month after surgery for all the outcomes, except for isometric knee extension, which should receive special attention.

## Figures and Tables

**Table 1:** Descriptive statistics of socio-demographic data.

Measures	Mean ± SD or (%) (*n* = 31)
Age	70.9 ± 10.93
BMI	26.12 ± 2.75
Gender	
Male	20 (65)
Female	11 (35)
Tumor stage T	
1	9 (29)
2	7 (23)
3	10 (32)
4	3 (10)
Unknown	2 (6)

Values are presented as mean ± standard deviation or number (%). BMI: Body Mass Index.

**Table 2:** Differences in clinical data at the different time points: preoperative one month (T1), postoperative three days (T2) and postoperative one month (T3).

	T1	T2	T3	Mean Difference T1–T2	Mean Difference T1–T3	Mean Difference T2–T3
SPPB score	10.07 ± 2.97	8.21 ± 3.9	9.64 ± 2.41	1.79	0.38	−1.42
Timed up and Go (s)	9.38 ± 5.91	9.49 ± 6.68	9.51 ± 5.39	0.12	−0.07	−0.13
Handgrip strength (kg)	26.77 ± 11.64	22.24 ± 10.66	25.3 ± 11.66	4.56 *	1.47	−3.09
Knee extension strength (kg)	22.31 ± 12.52	14.92 ± 10.14	17.38 ± 10.35	7.07 *	4.39 *	−2.68
Knee flexion strength (kg)	11.95 ± 5.55	13.23 ± 12.44	12.24 ± 5.87	−1.07	−0,28	0.79
Hip abduction strength (kg)	11.54 ± 10.65	8.05 ± 4.13	9.87 ± 4.53	3.12	1.41	−1.7 *
Hip adduction strength (kg)	10.55 ± 4.7	8.03 ± 4.32	10.35 ± 4.61	2.11	0.08	−2.03
Ankle dorsal flexion strength (kg)	9.42 ± 5.03	7.94 ± 3.04	8.89 ± 3.46	1.34	0.32	−1.02
Ankle plantar flexion strength (kg)	19.6 ± 17.55	11.57 ± 6.47	14.55 ± 9.61	7.43	4.82	−2.61
30 s Chair Stand Test (n° reps)	17.38 ± 8.16	12.54 ± 7.81	17.85 ± 8.58	4.61 *	−0.52	−5.13 *

* *p* < 0.05. SPPB: Short Physical Performance Battery.

**Table 3:** Differences in clinical data at the different time points: preoperative one month (T1), postoperative three days (T2) and postoperative one month (T3).

	T1	T2	T3	Mean Difference T1–T2	Mean Difference T1–T3	Mean Difference T2–T3
EQ-5D index score	0.87 ± 0.15	0.66 ± 0.21	0.79 ± 0.19	0.2 *	0.06	−0.13 *
QLQ-C30 global score	64.44 ± 26.62	53.88 ± 20.13	60.62 ± 29.65	10.88	6.16	−4.71
QLQ-C30 summary score	81.76 ± 18.89	69.91 ± 19.84	76.03 ± 14.99	10.64 *	4.11	−6.53

* *p* < 0.05. QLQ-C30: Cancer Quality of Life Questionnaire.

## Data Availability

Data can be available upon reasonable request to the corresponding author.
